# RAPA-ConvNets: Modified Convolutional Networks for Accelerated Training on Architectures With Analog Arrays

**DOI:** 10.3389/fnins.2019.00753

**Published:** 2019-07-30

**Authors:** Malte J. Rasch, Tayfun Gokmen, Mattia Rigotti, Wilfried Haensch

**Affiliations:** IBM Research AI, Mathematics of AI, Yorktown Heights, NY, United States

**Keywords:** resistive cross-point devices, analog computing, machine learning, emerging technologies, convolutional networks, hardware acceleration of deep learning

## Abstract

Analog arrays are a promising emerging hardware technology with the potential to drastically speed up deep learning. Their main advantage is that they employ analog circuitry to compute matrix-vector products in constant time, irrespective of the size of the matrix. However, ConvNets map very unfavorably onto analog arrays when done in a straight-forward manner, because kernel matrices are typically small and the constant time operation needs to be sequentially iterated a large number of times. Here, we propose to parallelize the training by replicating the kernel matrix of a convolution layer on distinct analog arrays, and randomly divide parts of the compute among them. With this modification, analog arrays execute ConvNets with a large acceleration factor that is proportional to the number of kernel matrices used per layer (here tested 16-1024). Despite having more free parameters, we show analytically and in numerical experiments that this new convolution architecture is self-regularizing and implicitly learns similar filters across arrays. We also report superior performance on a number of datasets and increased robustness to adversarial attacks. Our investigation suggests to revise the notion that emerging hardware architectures that feature analog arrays for fast matrix-vector multiplication are not suitable for ConvNets.

## 1. Introduction

Training deep networks is notoriously computationally intensive. The popularity of ConvNets is largely due to the reduced computational burden they allow thanks to their parsimonious number of free parameters (as compared to fully connected networks), and their favorable mapping on existing graphic processing units (GPUs; Chetlur et al., 2014).

Recently, speedup strategies of the matrix multiply-and-accumulate (MAC) operation (the computational workhorse of deep learning) based on mixed analog-digital approaches has been gaining increasing attention. Analog arrays of non-volatile memory provide an in-memory compute solution for deep learning that keeps the weights stationary (Yang et al., 2013; Fumarola et al., 2016). As a result, the forward, backward and update steps of back-propagation algorithms can be performed with significantly reduced data movement. In general, these analog arrays rely on the idea of implementing matrix-vector multiplications on an array of analog devices by exploiting their Ohmic properties, resulting in a one-step constant time operation, i.e., with execution time *independent* of the matrix size (up to size limitations due to the device technology; Gokmen and Vlasov, 2016).

Matrix-*matrix* multiplications can harness this time advantage from analog arrays, but since they are implemented as a sequence of matrix-vector products, their execution time is proportional to the number of such products. In other words, the time required to multiply a matrix on an analog array of size *n*_*o*_ × *n*_*s*_ with an input matrix of size *n*_*s*_ × *n*_*p*_ is not proportional to the overall amount of compute (∝*n*_*o*_*n*_*s*_*n*_*p*_, as for conventional hardware; He and Sun, 2015), but instead only scales linearly with the number of columns of the input matrix *n*_*p*_ and is invariant with respect to the size of the matrix stored on the analog array (*n*_*o*_ × *n*_*s*_).

These considerations indicate that ConvNets will not map favorably onto hardware architectures that use analog arrays for in-memory matrix-vector operations (Gokmen et al., 2017), as becomes clear when one formulates the convolution operation in terms of a matrix-matrix product (see section 2.1 for a detailed derivation). It turns out that kernel matrices (obtained by flattening and stacking convolution filters), are typically small, corresponding to a small size of the analog *n*_*o*_ × *n*_*s*_-array. More crucially, matrix-vector products need to be iterated *n*_*p*_ times (the number of image patches), which is proportional to the total number of pixels in the input image and can thus be very large, particularly for early conv layers.

A common strategy to speed up training is to use data parallelism, where updates over large batches of data are computed in parallel on independent computing nodes and then averaged (e.g., You et al., 2017). However, this is not a practical solution to speed up training on analog arrays, since weight updates are computed only implicitly on stationary weights in non-volatile memory and are thus not directly accessible for averaging (Gokmen and Vlasov, 2016).

**Figure 1:**
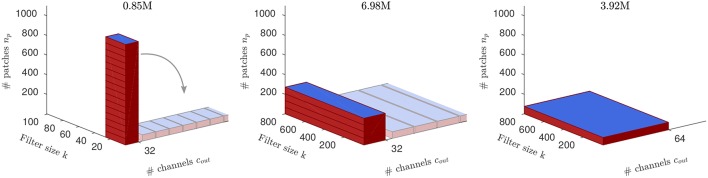
The amount of compute for the example ConvNet (respective for the 3 layers). Blue areas (*k* × *c*_out_) indicate the size of the kernel matrices. Computing time for analog arrays is proportional only to *n*_*p*_ and peaks at the first layer, while the amount of compute is *O*(*n*_*p*_*kc*_out_) (the volume of the red cuboid; MACs in titles) and peaks at the second layer. For each layer, our approach distributes the compute onto multiple replica of the kernel matrix residing on distinct arrays (“tiles”), indicated as tilings of the red cuboids into *n*_*t*_ = (16, 4, 1) small boxes, respectively. Since tiles are trained independently and in parallel, the compute time on analog arrays effectively becomes constant across layers (same height across layers; note, however, that the number of output channels of the convolution does not change). Our tiling schemes refer to the way individual image patches are assigned to the tiles.

Here, we propose a simple solution to accelerate ConvNets on systems with analog arrays, which we call RAPA Convolution (for *Replicated Arrays with Permuted Assignment*). The main idea is to use model parallelism to reduce the overall computation *time* on analog arrays (but not the *amount* of computation, as done e.g., in Figurnov et al., 2016). Concretely, we propose to replicate the kernel matrix onto *n*_*t*_ separate analog arrays (“tiles”), and to distribute the compute equally among the tiles (see Figure 1). When this architecture proposed for analog arrays is simulated on conventional hardware (as we do here), it is equivalent to learning multiple kernel matrices independently for individual conv layer. Thus, output pixels of the same image plane will be in general convolved with different filters. Note that we do not explicitly force the kernel matrices to be identical, which would recover the original convolution operation.

In this study, we simulate the training of RAPA ConvNets in order to validate the effectiveness of different ways to distribute the compute among the tiles and show that it is possible to achieve superior performance to conventional ConvNets with the same kernel matrix sizes. We further prove analytically in a simplified model that for a random assignment of compute to tiles, our architecture is indeed implicitly regularized, such that tiles tend to learn similar kernel matrices. Finally, we find that the RAPA ConvNet is actually more robust to white-box adversarial attacks, since random assignment acts as a “confidence stabilization” mechanism that tends to balance overconfident predictions.

### 1.1. Previous Work

Training of ConvNets with analog arrays has been previously investigated by Gokmen et al. (2017). However, that study focused on the effects of device inaccuracies in the analog arrays on the final classification performance, and did not investigate how to accelerate the run time of ConvNets by algorithmic changes, which is our focus here. To our knowledge, no previous work has proposed an implementation of ConvNets that harnesses the favorable scaling properties of analog arrays for inference and training. However, although proposed in a different context, some previous approaches share some similarities to ours from an algorithmic perspective. “Tiled convolutions” by Ngiam et al. (2010) are a special case of our algorithm, where multiple kernel matrices are used to compute pixels on a regular grid (instead of random assignments). “Perforated convolutions” by Figurnov et al. (2016), where some patches in the convolution operation are dropped to accelerate run time on conventional GPUs, are also related to our proposal. We therefore include both methods in our experiments comparing in detail these approaches with ours.

### 1.2. Analog Arrays

**Figure 2:**
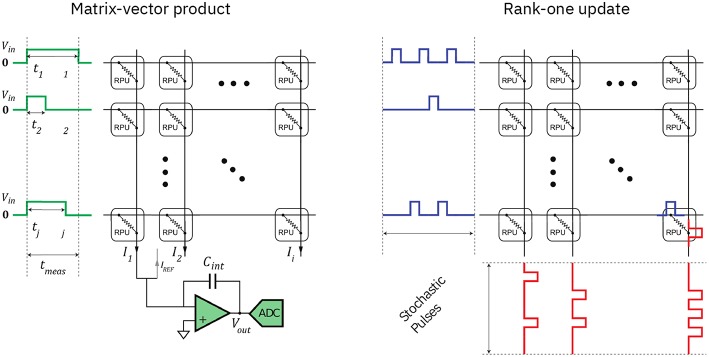
Computing matrix-vector multiplications and rank-one updates with an analog arrays using Ohm's property (adapted with permission from Gokmen and Vlasov, 2016). **(Left)** An input vector is encoded as a sequence of voltage signals and is applied to the weights, which are represented by the conductivity of the crossbar elements (RPU: resistive processing unit), resulting in a series of multiply-and-accumulate operations, whose results are represented by the output currents. **(Right)** A parallel rank-one update of all the matrix elements can be achieved by application of random trains of voltage pulses at both ends of the array. If each weight is being updated only if pulses coincide on both terminals of the corresponding cross-point, the resulting update will on average coincide with the outer product between the vectors encoding the pulse probabilities (see Gokmen and Vlasov, 2016 for further details).

Currently, a number of analog array technologies are under active development (Yang et al., 2013; Fumarola et al., 2016; Gokmen and Vlasov, 2016; Burr et al., 2017; Ambrogio et al., 2018), based on different device materials as candidates for the implementation of the switching elements encoding the modifiable synaptic weights (Burr et al., 2017). While the exact detailed training dynamics and operations at inference time depend on the type of device materials implementing the weights (Gokmen and Vlasov, 2016), the main scaling properties of analog arrays are independent of the underlying technology. In particular, the fact that a matrix-vector multiplication (during the forward or backward pass) and a rank-one update (weights update) can be performed as single step operations, i.e., with running time independent of the size of the matrix, is a general property of analog arrays. Figure 2 illustrates how these constant scalings are achieved by virtue of *Ohm's law* and using stochastic pulse sequences (see Gokmen and Vlasov, 2016 for details).

## 2. Methods

### 2.1. Convolution With Replicated Kernel Matrices

Following common practice (e.g., Chetlur et al., 2014), the convolution of a filter of size *k*_*h*_ × *k*_*w*_ over an input image of size *h* × *w* × *c*_in_ can be formulated as a matrix-matrix multiplication between an *n*_*p*_ × *k* *im2col* matrix *I*, constructed by stacking all *n*_*p*_ (typically overlapping) image patches **b**_*i*_ of size *k*_*h*_ × *k*_*w*_ × *c*_in_ in rows of length *k* = *k*_*h*_*k*_*w*_*c*_in_. We can then write $I={\left({\text{b}}_{1},\ldots ,{\text{b}}_{n_{p}}\right)}^{T}\equiv {\left({\text{b}}_{i}^{T}\right)}_{i\in \left\{1,\ldots ,n_{p}\right\}}$. The matrix *I* is then multiplied by the *k*×*c*_out_ kernel matrix *K*, where *c*_out_ is the number of output channels (i.e., the number of filters). The result *M* = *IK* is of size *n*_*p*_×*c*_out_, and is finally reshaped to a tensor with size $\tilde{h}\times \tilde{w}\times c_{\text{out}}$, to reflect the original image content.

In most ConvNets, conv layers are alternated with some form of pooling layers, that reduce the spatial size typically by a factor of 2 (the pool stride; Gu et al., 2018). Thus, for the next convolutional layer, *n*_*p*_ is reduced by a factor of 4 (square of the pool stride). On the other hand, because output channels become the input channels to the following layer, the size of *K* changes as well (see Figure 1).

Our approach to parallelize the compute on analog arrays consists in using *n*_*t*_ kernel matrices *K*_*j*_ instead of just one *K* for a given conv layer, and distributing the patches **b**_*i*_ equally among them, so that at any given time *n*_*t*_ matrix-vector products can be processed in parallel. Each of the *n*_*p*_ patches is assigned to exactly one subset *S*_*j*_⊂{1, …, *n*_*p*_} (all of roughly equal size, |*S*_*j*_|≈*n*_*p*_/*n*_*t*_), and the individual array tiles effectively compute the sub-matrices $M_{j}=I_{j}K_{j}={\left({\text{b}}_{l}^{T}\right)}_{l\in S_{j}}K_{j}$. How the image patches are divided into the subsets *S*_*j*_ is what we call “tiling scheme” (see below).

The final result is then obtained by re-ordering the rows according to their original index. In summary, with *s*_*l*_ = *j* if *l*∈*S*_*j*_, we can write $M_{\text{tiled}}={\left({\text{b}}_{l}^{T}K_{s_{l}}\right)}_{l\in \left\{1,\ldots ,n_{p}\right\}}$. Note that if all *K*_*j*_ are identical, the tiled convolution trivially recovers the original convolution. If we assume that each kernel matrix *K*_*j*_ resides on a separate analog array tile, and all resulting *I*_*j*_*K*_*j*_ operations can be computed in parallel, the overall computation is sped up by a factor of *n*_*t*_ (neglecting the effort of the assignment, since that can be done efficiently on the digital side of the mixed analog-digital system).

**Figure 3:**
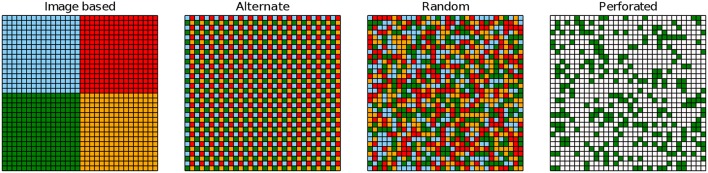
Illustrated is the output of a conv layer for different tiling schemes (*n*_*t*_ = 4, *c*_out_ = 1). Each output pixel might be computed with a kernel matrix from a different array tile (colors; white means zeros).

However, if all *K*_*j*_ are learned independently and without explicit synchronization (a prerequisite for embarrassingly parallel execution) filters corresponding to the same output channel might in general be non-identical, which implies that *M*_tiled_ ≠ *M*. Thus, learning all *K*_*j*_ in parallel might negatively impact accuracy. In the following, we test how different tiling schemes affect the overall accuracy. We use the following schemes (compare to Figure 3).

#### 2.1.1. Image-Based Tiling

This tiling scheme consists in collecting all patches that contain pixels from a particular image region into a common subset *S*_*j*_. If the image is a square with sides of length *n* and the number of tiles *n*_*t*_ is a square number, $n_{t}=q^{2}$, the patch **b**_*i*_ centered at pixel position (*x*_*i*_, *y*_*i*_) with *x*_*i*_, *y*_*i*_∈{0, …, *n*−1} is assigned to the subset *S*_*s*_*i*__, with $s_{i}=\left\lfloor \frac{qx_{i}}{n}\right\rfloor +q\left\lfloor \frac{qy_{i}}{n}\right\rfloor +1.$. Note that image patches at the border will generally contain pixels from the neighboring regions. We thus call this scheme “image w/overlap.” Alternatively, the pixels from other regions can be set to zero (as if padded in case of separate sub-images), and we call this scheme “image w/pad.”

#### 2.1.2. Alternate Tiling

If the image is again a square and $n_{t}=q^{2}$, one could put image patches that are neighboring to each other into different subsets, so that neighboring image patches are assigned to alternate tiles. Specifically, *s*_*i*_ = (*x*_*i*_ mod *q*)+*q*(*y*_*i*_ mod *q*)+1. This tiling is similar to the “tiled convolution” approach suggested by Ngiam et al. (2010) as a way to improve the learning of larger rotational and translational invariances within one convolutional layer.

#### 2.1.3. Random Tiling

An alternative way of distributing *n*_*p*_ image patches onto *n*_*t*_ kernel matrices, is to let the *S*_*j*_ be a random partition of the set {1, …, *n*_*p*_}, with each of the *S*_*j*_ having (roughly) the same size. We investigate two cases: one where the partition is drawn once at the beginning and fixed the remainder (“random fixed”), and the case where we sample a new partition for each train or test image (“random”).

#### 2.1.4. Perforated Convolution

An alternative way to speed up convolutions, is to simply train a single kernel matrix with only a fraction *n*_*p*_/*n*_*t*_ of the data (Figurnov et al., 2016). As a result many output pixels will have zero value. Thus, in this scheme we randomly draw a subset *S* of *n*_*p*_/*n*_*t*_ indices and set the rows for which *i* ∉ *S* to **0**, as described for Ngiam et al. (2010). We resample *S* for each image during training and use all available image patches during testing. Note that in this scheme only a single kernel matrix is used.

### 2.2. Network Parameters Used in the Experiments

We perform a battery of proof of concept experiments using a small standard ConvNet on 3 datasets: CIFAR-10, CIFAR-100 (Krizhevsky and Hinton, 2009), and SVHN (Netzer et al., 2011). The network^1^ consists of three conv layers with kernel size 5 × 5, and intermediate pooling layers of stride 2. We tried several options for the first two pooling layers (see Results section), whereas the last pooling layer is fixed to an average pooling. The first two conv layers are followed by lateral response normalization, and the last conv layer is followed by a fully connected layer. We also use a very small weight decay (0.0001 times the learning rate) if not otherwise stated and mini-batch of 10, train for > 400 epochs and report the minimal test and train errors (as average over five consecutive epochs). The learning rate λ is annealed in a step-wise manner every 25, 100, or 300 epochs with a factor λ_γ_, and multiple settings were tested (settings were identical for experiments that were directly compared). If multiple runs on the datasets were made with different learning rate settings, we report the best test error. We found that λ = 0.005 for no tiling, and λ = 0.025 for tiling, seemed to work best (with λ_γ_ = 0.1, step size 300 epochs, trained for 700 epochs). Note that the number of updates is effectively reduced per array tile, which can be in part compensated by increasing the learning rate. We additionally use a constant “warm up” period of 1 or 5 epochs with a learning rate reduced by a factor of 20.

The output channel setting of the network is 32, 32, 64 for the conv layers, respectively. Thus, for CIFAR-10 the network has 79328 weights (including biases) only in the conv layers. For tiling with *n*_*t*_ = (16, 4, 1) tiles, the number of convolutional weights are increased to 192,704. To compare this against a network of roughly the same number of weights, we increase the number of channels for the non-tiled network to 54, 64, 64, which yields 193032 weights (“enlarged” network). However, note that for this larger network the amount of compute is actually increased, whereas the amount of compute of the tiled network is identical to the original ConvNet.

For training we used standard stochastic gradient descent with 32 bit floating point precision if not otherwise stated. We use moderate image augmentations (mirroring and brightness changes) if not stated otherwise. All experiments are implemented in the Caffe2 framework (Jia et al., 2014) (using custom C++/CUDA operators, where necessary).

Finally, in addition to the usual pooling methods (max-pooling, average-pooling and stochastic pooling, reviewed e.g., in Gu et al., 2018), we also applied mixed pooling to get the benefits of both max and average pooling. In particular, similar to Yu et al. (2014), we use a learnable combination of average and max-pooling, with mixture parameters per channel α_*k*_ ∈ [0, 1]. To enforce these parameter limits, we set $\alpha_{k}\equiv \frac{1}{1+e^{\mu \beta_{k}}}$ and train the β_*k*_ with μ = 10 fixed. Initial values are β_*k*_ = 2/μ to ensured a bias toward max-pooling, which works best on the datasets used here.

## 3. Results

Our aim here is to systematically quantify the relative impact of the convolutional tiling architecture on performance, not to reach state-of-the-art accuracy on the tested datasets. We therefore examine a relatively small standard ConvNet with 3 conv layers (see section 2.2).

**Figure 4:**
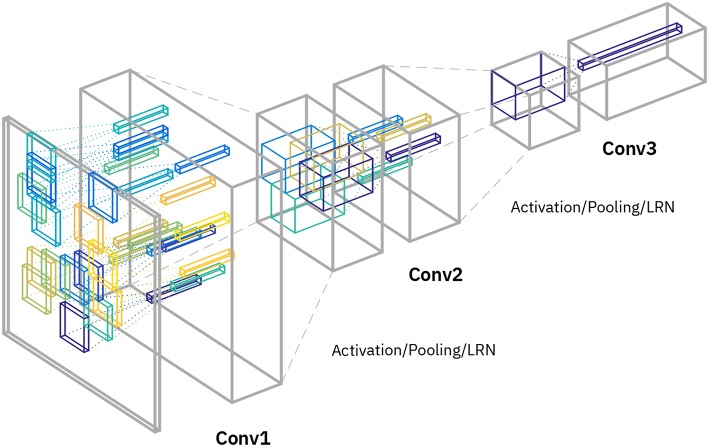
Network architecture for “random” tiling. Three convolution layers are interleaved with pooling and activation. Note that only the convolutional layers are displayed, and the final stages (including the final pooling layer and the fully connected layer) are omitted in the diagram, since they identical to the original network. The first conv layer (“Conv1”) uses 16 different kernel matrices (indicated with the different colors) and the image patches are randomly distributed among these (with a new random permutation drawn for each image). The second conv layer (“Conv2”) uses 4 different kernel matrices (indicated with colors) and patches are similarly randomly distributed among those. The last conv layer (“Conv3”) uses just 1 kernel matrix as for normal conv layers. The number of replicated kernel matrices per layer are chosen to match computing times in each layer on analog arrays.

As described, only the number *n*_*p*_ of input patches per layer determines the run time on analog arrays. We thus divide the compute of each conv layer onto *n*_*t*_ array tiles, so that the number of image patches per tile, *n*_*p*_/*n*_*t*_, is constant. Since we have *n*_*p*_ = (1024, 256, 64) across the three layers, we use *n*_*t*_ = (16, 4, 1) tiles for the 3 conv layers, respectively. Note that this architecture achieves perfect load-balancing across the conv layers, because each tile in the network learns a separate kernel matrix using 64 image patches per image. See Figure 4 for an illustration of this architecture in the case of *random tiling*.

### 3.1. Main Experimental Results

We tested the performance of this setup on the three datasets with and without tiling, and compared different tiling schemes using floating point (FP) precision (see Table 1, FP columns). The main results from these experiments are: (1) *Random tiling* achieves the best performance among all tiling schemes; (2) Across datasets, *random tiling* comes close or actually beats the regular ConvNet with no tiling; (3) Simply subsampling the input images is not sufficient to explain the high performance of *random tiling*, since the *perforated scheme* generally performed poorly.

**Table 1:** Best test (train) error [%] (determined by an average of 5 consecutive training epochs) for different tiling schemes across datasets.

**Tiling \ Data**	**CIFAR-10**		**SVHN**		**CIFAR-100**	
**Precision**	**FP**	**RPU**	**FP**	**RPU**	**FP**	**RPU**
Random fixed	24.7 (4.0)	24.2 (3.5)	11.4 (2.4)	11.3 (0.3)	55.6 (19.1)	59.7 (37.4)
Random	17.3 (6.8)	19.1 (13.8)	7.3 (4.2)	7.8 (5.8)	48.4 (31.8)	54.1 (45.6)
Voting	**16.3** (6.8)	**17.1** (13.8)	**6.5** (4.2)	**6.2** (5.8)	**47.7** (31.8)	**51.3** (45.6)
Image w/overlap	22.6 (0.1)	22.5 (3.1)	10.4 (3.1)	10.4 (0.7)	53.1 (35.3)	58.0 (37.5)
Image w/pad	24.1 (0.7)	25.5 (9.2)	11.3 (6.2)	12.5 (2.6)	54.3 (29.0)	60.6 (43.9)
Alternating	21.1 (4.1)	20.4 (4.6)	9.4 (3.1)	9.4 (1.2)	52.3 (19.0)	54.4 (36.3)
No tiling	18.5 (6.2)	19.1 (9.3)	9.1 (3.0)	9.3 (3.0)	48.3 (26.6)	52.5 (39.1)
Perforated	27.2 (22.2)	32.6 (29.6)	8.9 (11.6)	11.1 (18.4)	63.9 (50.4)	76.5 (62.3)
Enlarged	16.1 (0.0)	17.5 (5.1)	9.1 (0.7)	8.7 (0.5)	47.0 (20.5)	50.1 (30.5)

*Best tiling method is marked in bold. Note that random tiling with majority vote is consistently the best tiling method and matches or reaches superior performance to the original non-tiled ConvNet. The last three rows are control conditions without replication of the kernels. FP columns: floating point results. RPU columns: RPU hardware-aware simulation that includes various device-to-device variation, noise and imprecision, as well as a simulated weight update with finite pulse widths. Note that device switching behavior is assumed to be noisy but ideally balanced on average (RPU model as described in Rasch et al., 2019). Results are qualitatively similar to the floating point simulation, except that the finite weight states, limited precision, and noise generally cause a slight drop in performance for both original non-tiled ConvNet and the tiled versions, in particular for the more challenging CIFAR-100 dataset. For RPU simulations, we turned off the weight decay, because it would not be straight-forward to implement in RPU hardware. Parameters: learning rate varied from λ = 0.0005 to λ = 0.05 for each condition, reduced every 300 epochs by 10, and 700 training epochs (best result taken)*.

Our experiments show that random tiling matches or even outperforms the original network (see Table 1, “random” vs. “no tiling”). The performance of the random tiling network in Table 1 (“random”) is obtained by sampling only one random assignment of patches to tiles during test. However, for each test image, we can also generate multiple predictions, each generated by a different random assignment, and take as final output the majority vote of all predictions (similarly e.g., to Graham, 2014). We find that the performance gain due to majority voting can be considerable (see “random voting” column in Table 1; voting over 9 predictions).

That a tiled network architecture could in principle outperform the original ConvNet, can be understood when considering that replicating kernel matrices onto multiple tiles effectively increases the number of free parameters in the network (here by about a factor of 2.5, see section 2.2). Thus an approximate upper limit of the performance of the tiled networks can be obtained by comparing the performance to a non-tiled network with a similar number of free parameters arranged in conventional fashion (by increasing the number of channels per layer). It is important to note in this comparison that, despite having more free parameters, the amount of compute (i.e., the number of MAC operations) of the tiled network is nevertheless identical to the original non-tiled ConvNet. In contrast, if the number of channels in the original network is enlarged, the number of MACs, and thus the run time on a digital processor, increases as well.

We do indeed find that this enlarged network achieves a performance comparable or better than the random tiling network with voting (compare Table 1 “random voting” with “no tiling enlarged”) in both CIFAR datasets. In the SVHN dataset, however, the enlarged network actually still performs worse than the random tiling method, showing that the randomness in the tiling network helps to regularize its increased set of parameters very well (see also theoretical analysis below).

### 3.2. Simulation With Reduced Precision on Simulated RPU Arrays

Analog resistive crossbar arrays suffer from a number of inaccuracies and noise sources due to analog circuitry and device material non-idealities, as has been previously investigated extensively (Gokmen and Vlasov, 2016; Gokmen et al., 2017, 2018; Haensch et al., 2019). Our comparison of the tiling networks above was done in floating point precision. Thus, while we do not expect that tiling networks solve any known performance impacts due to hardware non-idealities, it is nevertheless important to assert that they do not introduce new challenges and that our tiling comparison results are qualitatively reproduced when simulated in a more analog hardware realistic fashion.

We thus repeated the analysis by using the same RPU array model and compensatory measures described in Rasch et al. (2019), which is based on the RPU model and hardware specifications from Gokmen and Vlasov (2016), Gokmen et al. (2017), and Gokmen et al. (2018), except that the number of device states are increased by 4 (i.e., minimal update pulse width Δ*w*_min_ = 0.00025, compare to Gokmen and Vlasov, 2016) and that the switching behavior of the devices is ideally symmetric on average for simplicity. Note that this RPU array model has saturating weight bounds (with device-to-device variation), stochastic update pulse train generation (see Figure 2), device-to-device and cycle-to-cycle variation in the update pulse widths, cycle-to-cycle additive noise, and limited dynamic input-output range, among others (see e.g., Gokmen et al., 2018 for a detailed description), and thus models many of the inaccuracies and noise sources that plague analog arrays. We further assume that each kernel replica occupies a separate crossbar array (of sufficient size and exact dimensions), each having exclusive access to analog-digital converters (ADC and DAC resolution are assumed to be 9 and 7 bit, respectively) and have the same hardware specifications.

With this RPU array model, we repeated the comparison of the tiling networks across all datasets (see Table 1, “RPU” columns). We find that analog imprecisions and variations introduce an increase in test error of only up to a few percentage points compared to training in floating point precision (Table 1, “FP” columns). As can be expected, error increases mostly for the more challenging dataset CIFAR-100. While the exact test error achieved by the RPU simulations will depend on the device model chosen (e.g., the number of material states, switching behavior), the ranking between the tiling methods as described above for FP precision is robustly preserved, with random tiling with voting still showing up as the best method for all datasets, matching or beating the performance of the original ConvNet.

### 3.3. Increasing Number of Tiles

An advantage of random tilling over alternating and image-based tiling is that it is straightforward to use with an arbitrary number of tiles per layer, that does not for instance necessarily correspond to a square number (see Methods for definition of the tiling methods). Thus one can easily increase the number of tiles per layer to achieve even higher theoretical run-time on analog RPU arrays. Note that it is advisable to use a number of tiles per layer that balances the number of patches processed by each tile. Since the number of patches reduces by 4 per layer in our example networks, a favorable tile number setting would be e.g., *n*_*t*_ = (64, 16, 4), respectively, for the three layers, which then would theoretically run 64 × faster than the original ConvNet on analog RPU arrays, with perfect load-balancing among the convolutional layers.

**Figure 5:**
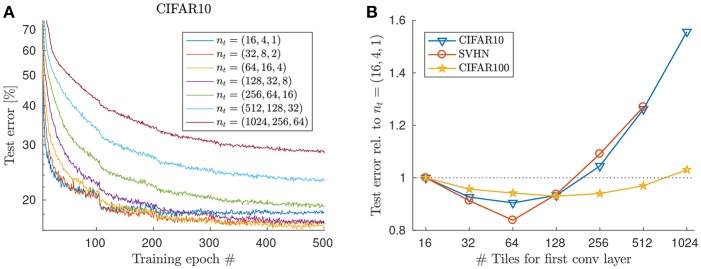
Increasing the tile number in case of the random tiling method. **(A)** Test error (with majority voting from 9 predictions) is plotted vs. training epochs for the CIFAR-10 dataset. Note that increasing number of tiles moderately beyond 16 results in even better test error, because of better regularization. However, learning is slower and very high tile numbers (> 128) fail to reach a competitive test error with this learning rate scheduling. **(B)** Best test error [relative to the random tiling network with *n*_*t*_ = (16, 4, 1)] across datasets. Parameters: λ = 0.05, halving the learning rate every 100 epochs. No weight decay.

To test the effect of increasing the number of tiles in the case of the random tiling method, we increased the number of tiles proportionally in each layer, up to reaching 1,024 tiles in the first layer, 256 in the second, and 64 in the third layer (see Figure 5A). We don't notice any deterioration of test performance due to overfitting compared to the 16 tiles case up until 128 tiles in the first layer. Interestingly, test error actually improves when the increase in number of tiles is moderate (Figure 5B), indicating that the regularization property of the architecture is enough to prevent overfitting, presumably because the 3rd conv layer now also uses multiple kernel replica.

Unsurprisingly though, increasing the number if tiles even further starts to hurt test performance. For instance, using more than 128 in case of CIFAR-10, results in an increase in test error, presumably because the number of samples per tile becomes too small and kernel replicas too noisy. We set the weight decay to zero in this comparison, to prevent the less frequently updated weights (in case of larger tile numbers) to decay faster to zero. Whether e.g., adjusting the learning rate or weight decay with the number of tiles per layer or other compensatory measures might recover some of the lost accuracy for very high tile numbers, is a direction for further research.

### 3.4. Regularization and Filter Similarity Across Tiles

Since replicated kernel matrices are trained independently, it is interesting to examine the similarity of the filters at the end of training, which would hint at the degree of regularization of the replicated kernel across tiles. Note that only for identical filters across tiles, the original convolution is recovered.

**Figure 6:**
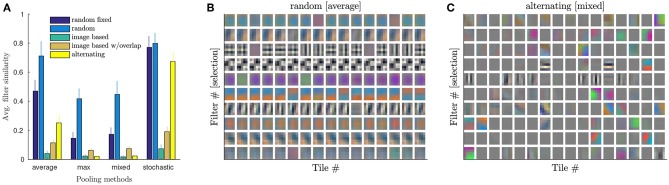
Similarity of learned kernel matrices *K*_*j*_ for the first convolution. **(A)** Similarity of *K*_*j*_ for different tiling methods and different pooling in case of CIFAR-100^+^.Similarity is computed by averaging the correlation coefficients between all pairs of corresponding filters across filter replicas and than averaging over all filters per layer. Plotted is the average similarity over the tiled conv layers. Compare to Table 2 for corresponding performances. **(B)** Selection of 10 out of 32 filters (rows of *K*_*j*_; reshaped) for all array tiles for average pooling and random tiling method trained on CIFAR-100^+^. Filters are very similar across tiles. **(C)** Like B but for mixed pooling and alternating tiling. Test error (42.4%) is similar to **(B)** (43.2% with voting). However, filters are very different across the 16 tiles.

For the random tiling scheme, where the input distribution across tiles should tend to be very similar on average across training epochs, different replicated filters are predicted to be more similar. This should however not be the case for other tiling schemes, where the mapping of image regions to tiles is fixed throughout training. Indeed, if we quantify the average similarity of the learned filters across array tiles (computing the average correlation coefficients between all pairs across tiles, averaged over output channels) we find low values for all tiling schemes trained with max-pooling, except for the random tiling scheme (see Figure 6A, max pooling).

In general, two main factors tend to implicitly force kernel matrices to become similar during training: (a) input patch similarity across tiles, and (b) specificity of the error-signal across tiles.

To test these effects we run additional experiments (with floating point precision) on strongly augmented datasets with different pooling methods.

#### 3.4.1. Explicit Regularization by Strong Data Augmentation

We further analyze the self-regularizing property that tiling confers to our architecture. Since regularization helps mitigate overfitting when the number of model parameters is large compared to the training dataset, a natural way of doing this is to quantify the test performance after enlarging the training dataset size. For this we use data augmentation by random scaling and jittering of the input images, a common regularization technique that is very effective in dealing with small datasets in object recognition tasks (e.g., see Perez and Wang, 2017 for a recent comparison). When we apply such a strong data augmentation (random scaling by up to 130%, scale jittering, and random cropping), we find that test errors are indeed reduced significantly across datasets and methods (see Table 2, column “M”; we also varied pooling methods, see below).

**Table 2:** Best test error [%] (determined by an average of 5 consecutive training epochs) for tiling schemes when datasets are strongly augmented.

**Tiling \ Data**	**CIFAR-10**^**+**^				**SVHN**^**+**^				**CIFAR-100**^**+**^			
**Pooling**	**M**	**A**	**X**	**S**	**M**	**A**	**X**	**S**	**M**	**A**	**X**	**S**
Random fixed	74.1	85.1	17.3	57.4	35.8	28.5	7.0	37.0	66.6	77.7	43.9	75.0
Random	17.5	18.3	17.6	24.3	7.6	8.3	7.4	14.0	44.0	45.3	44.2	51.3
Voting	**15.8**	17.4	**15.7**	21.3	**6.0**	7.3	**5.9**	10.3	**40.6**	43.2	**41.2**	47.4
Image w/overlap	17.6	19.0	17.5	24.6	7.2	7.9	7.2	11.5	45.8	46.5	45.7	53.0
Image w/pad	20.9	22.5	20.6	29.9	9.4	10.3	9.2	19.0	48.5	49.5	48.6	57.5
Alternating	79.8	87.9	**15.3**	57.8	31.3	45.1	**6.7**	39.6	64.8	64.2	**42.4**	85.0
No tiling	16.0	16.5	15.1	22.9	6.7	7.5	7.0	10.9	43.4	43.1	42.6	50.0
Perforated	33.6	35.2	31.0	54.9	13.6	15.1	12.5	44.6	65.0	65.2	67.3	92.4
Enlarged	13.0	14.1	13.0	18.9	5.7	6.4	6.0	9.0	40.5	40.9	40.3	46.2

Separated for different pooling methods (M: max pooling, A: average pooling, X: mixed pooling, S: stochastic pooling) for the first two layers (last is average pooling). The three best tiling and pooling combinations per dataset are marked in bold. The last 3 rows are comparisons with conventional non-tiled networks. Parameters: λ = 0.025, reduced every 300 epochs by 10, and 700 training epochs. Floating point precision is used.

Indeed, our results using the strongly augmented datasets show that other tiling methods are now much closer in performance in comparison to the random tiling method (e.g., in Table 2 compare “random” vs. “random fixed” for mixed pooling “X”). This is understandable, because now the input data is explicitly regularized, and the implicit regularization due to random tiling is less necessary. However, we nevertheless find that random voting is consistently the best among tiling methods (see Table 2 bold values) across datasets. Interestingly, with explicit regularization through data augmentation the alternating tiling method performs also very well, beating the other tiling methods without voting. However, the alternating tiling method only yields robust results in case of mixed pooling and diverges for other pooling methods in our experiments.

The original non-tiled ConvNet also gains in performance thanks to data augmentation, in particular for the more challenging CIFAR-100 dataset, where data is very limited and augmentation prevents overfitting. Typically, we find that the best tiling methods (random voting and alternating) still approximately match or outperform the original non-tiled ConvNet, but performance does not exceed in the enlarged ConvNet, which no longer suffers from overfitting (see Table 2).

Note, however, that when using analog arrays, the number of channels can be increased without increasing the run time (assuming sufficient device resources), because of the described scaling laws of analog arrays. Thus, one can always increase the number of channels of a tiled ConvNet on an analog arrays (up to the device resource limits) to increase the accuracy performance further without incurring a decrease in run time.

#### 3.4.2. Specificity of Error-Signal Across Tiles

To also investigate the effect of similarity of the error-signal received by different tiles, we conduct a series of experiments replacing the first two max-pooling layers with other types of pooling (average, mixed, or stochastic pooling, see later for explanation). The type of pooling operation determines how backpropagated errors are propagated to the inputs. In the case of average pooling (followed by ReLU), all tiles contributing to a positive pixel value in a pooling region will receive the same error signal, whereas for max-pooling only the output pixel with the maximal value per pooling region is selected and used to only update the one corresponding tile. A trade-off between these effects can be achieved by learnable mixture between max and average pooling which we call mixed pooling. Finally, we also tested stochastic pooling, where a random output pixel in the pooling region is selected, and thus all pixels (and the corresponding tiles) will receive similar updates on average over the training process.

We find that all pooling methods induce some degree of similarity in the case of random tiling (see Figure 6A). As expected, the highest similarity is for average and stochastic pooling, and tiling methods where a 2 ×2 pooling region typically contains pixels that are computed with different kernel replica (compare to Figure 3). Indeed, stochastic and random pooling also induce filter similarity in other tiling methods, in particular “random fixed” and “alternating.” However, both methods struggle to converge with these pooling methods (see Table 2), suggesting that some diversity among replicated kernel matrices might be advantageous. In Figures 6B,C, example filters are plotted for the first convolutional layer (having 16 tiles) in case of CIFAR-100 (strongly augmented). Both methods, random tiling with average pooling and alternating with mixed pooling, show similarly good test errors. However, the similarity of the kernels across tiles is strikingly different.

#### 3.4.3. Reduction of Tiled Network to the Original Architecture

It might be problematic for certain applications to retain multiple kernel matrices per conv layer. Thus, one might want to recover the original network, after benefiting from the training speedup of the tiled network.

If the filters are very similar (as with average or stochastic pooling, see Figure 6A) just taking a kernel matrix of any tile recovers the original convolution and with a performance close to that of the original network (see Table 3 “random,” column “A”).

**Table 3:** Best test error [%] when reducing the tiled network to the original convnet structure by forming a single kernel per layer from the replicas (best of following filter selection methods: norm-based, random tile, first tile, or random filters of any tile).

**Tiling \ Data**	**CIFAR-10**^**+**^				**SVHN**^**+**^				**CIFAR-100**^**+**^			
**Pooling**	**M**	**A**	**X**	**S**	**M**	**A**	**X**	**S**	**M**	**A**	**X**	**S**
Random fixed	72.2	83.7	32.5	51.3	48.8	34.8	22.8	23.5	80.6	89.9	66.4	70.6
Random	22.1	**20.1**	24.0	24.1	17.4	**9.4**	16.5	13.9	48.2	**47.9**	52.0	51.3
Image w/overlap	61.6	52.3	57.7	47.9	25.9	21.1	21.0	27.4	90.7	86.1	91.5	78.6
Image w/pad	74.5	60.9	71.5	53.7	59.1	45.9	51.0	36.9	92.8	87.8	92.3	82.2
Alternating	81.0	87.0	38.7	53.8	53.8	60.1	31.0	32.7	91.6	86.5	72.5	83.4

*Note that only random tiling with re-shuffling results in easy reduction to the original ConvNet, in particular when using average pooling. Some performance impact is however present, equaling approximately 2–5 test error percent points increase for the three datasets, compared to the results of directly training the non-tiled network (also with average pooling method; data from Table 2). Best tiling and pooling combination per dataset is marked in bold*.

Another way to reduce the tiled model for mixed or max-pooling, is to select among all replica the filters that most often “wins” the maximum pooling on the training set. These can then be combined to form a single kernel matrix. An alternative simpler way is to just select across tiles the filter with the highest norm, since that indicates a filter that is more often used and updated, and therefore less subject to the weight decay penalty.

We tested these reduction techniques and found only in case of random tilings a slightly worse but still acceptable test error of the reduced model when compared to the conventionally trained ConvNet (see Table 3). The reduction to the original network seems to work best for random tiling with average pooling, which has a good compromise between performance and kernel similarity. It needs to be investigated further whether a smarter kernel selection during reduction, possibly together with a short retraining process, could recover the full accuracy of the non-tiled ConvNet.

However, note, that reducing the random tiling network to the original architecture also removes the benefits of accelerated run time on analog arrays, the performance gain by majority voting, and the robustness to adversarial attacks (investigated below).

### 3.5. Theoretical Analysis: Implicit Regularization of Random Tiling

It is rather intriguing that our random tiling scheme achieves a performance that is comparable or even better than the standard ConvNet. One might have expected that as many as 16 replicated kernel matrices for one conv layer would have incurred overfitting. However, empirically we see that random tiling actually tends to display less overfitting than the standard ConvNet. For example for the SVHN data set in Table 1, we see that e.g., the enlarged standard ConvNet (no tiling) achieves a test error of 9.1% with a training error close to zero, while random tiling has a better test error rate of 7.3% (without voting) with higher training error (4.2%). In this section, we give a formal explanation of this phenomenon and show in a simplified model, a fully-connected logistic regression model, that replicating an architecture's parameters over multiple “tiles” that are randomly sampled during training acts as an implicit regularization that helps to avoid overfitting.

A logistic regression is a conditional distribution over outputs *y* ∈ {0, 1} given an input vector ***x*** ∈ ℝ^*d*^ and a set of paramters **θ** ∈ ℝ^*d*^. The exponential family distribution form of the logistic regression is

p(y|x,θ)=exp(y x·θ-A(x·θ)),

where *A*(*z*) ≡ −log(1−σ(*z*)) and σ(*z*) ≡ (1+exp(−*z*))^−1^ is the logistic function. Note that this expression is equivalent to the more common form *p*(*y* = 1|**x**, **θ**) = σ(**x**·**θ**). Training a logistic regression consists in finding parameters that minimize the empirical negative log-likelihood,

$$l_{x,y}\left(\theta \right)=-\log p\left(y|x,\theta \right),$$

over a given set of *N* training examples (**x**^*i*^, *y*^*i*^), resulting in the minimization of the loss:

$$L\left(\theta \right)=\overset{N}{\underset{i=1}{\sum }}l_{x^{i},y^{i}}\left(\theta \right).$$

We model random tiling by assuming that every parameter θ_*l*_ is being replicated over *n*_*t*_ tiles. Correspondingly, every time θ_*l*_ is being accessed, a parameter $\theta_{l}^{s_{l}}$ with *s*_*l*_ randomly sampled in {1, …, *n*_*t*_} is retrieved. We write $\theta^{s}\equiv {\left(\theta_{l}^{s_{l}}\right)}_{l}$ and **s** ≡ (_*s*_*l*_)*l*_. As a result training can be expressed as the minimization of the average loss,

$${\langle L\left(\theta^{s}\right)\rangle }_{s}=\overset{N}{\underset{i=1}{\sum }}{\langle l_{x^{i},y^{i}}\left(\theta^{s}\right)\rangle }_{s},$$

where the angular brackets 〈·〉_**s**_ indicate averaging over the process of randomly sampling every parameter θ_*l*_ from a tile *s*_*l*_. With the above, we get

$$\begin{array}{l} {\langle L\left(\theta^{s}\right)\rangle }_{s}=-\overset{N}{\underset{i=1}{\sum }}\left(y^{i}\;x^{i}\cdot \bar{\theta }-{\langle A\left(x^{i}\cdot \theta^{s}\right)\rangle }_{s}\right)\\ \;=L\left(\bar{\theta }\right)+R\left(\left\{\theta^{s}\right\}\right), \end{array}$$

where $\bar{\theta }$ is the vector whose components are the parameters averaged across tiles, i.e., ${\bar{\theta }}_{l}={\langle \theta_{l}^{s_{l}}\rangle }_{s}$, and

$$\begin{array}{l} R\left(\left\{\theta^{s}\right\}\right)=\overset{N}{\underset{i=1}{\sum }}\left({\langle A\left(x^{i}\cdot \theta^{s}\right)\rangle }_{s}-A\left(x^{i}\cdot \bar{\theta }\right)\right). \end{array}$$

The term *R*({**θ**^**s**^}) that falls out of this calculation has the role of a regularizer, since it does not depend on the labels *y*^*i*^. In a sense, it acts as an additional cost penalizing the deviations of the replicated parameters **θ**^**s**^ from their average value $\bar{\theta }$ across tiles. This tendency of the replicated parameters to move toward the mean counteracts the entropic pressure that training through stochastic gradient descent puts on the replica to move away from each other (see e.g., Zhang et al., 2018), therefore reducing the effective number of parameters. This implicit regularization effect explains why, despite the apparent over-parametrization due to replicating the parameters over tiles, our architecture does not seem to overfit more than its standard counterpart. It also explains the tendency of the tiles to synchronize causing the filters to become similar (Figure 6).

### 3.6. Robustness Against Adversarial Examples

We can gain further intuition on the role of the regularizer *R*({**θ**^**s**^}) by developing its first term as a Taylor series up to second order around $x^{i}\cdot \bar{\theta }$, analogously to what is done in Bishop (1995), Rifai et al. (2011), and Wager et al. (2013). This results in:

$$\begin{array}{l} R\left(\left\{\theta^{s}\right\}\right)\approx \frac{1}{2}\overset{N}{\underset{i=1}{\sum }}A^{\prime \prime }\left(x^{i}\cdot \bar{\theta }\right)\underset{l}{\sum }{\left(x_{l}^{i}\right)}^{2}\;{\text{Var}}_{s}\left(\theta_{l}^{s_{l}}\right)\\ \;=\frac{1}{2}\overset{N}{\underset{i=1}{\sum }}p_{i}\left(1-p_{i}\right)\underset{l}{\sum }{\left(x_{l}^{i}\right)}^{2}\;{\text{Var}}_{s}\left(\theta_{l}^{s_{l}}\right), \end{array}$$

where ${\text{Var}}_{s}\left(\theta_{l}^{s_{l}}\right)$ is the variance of the parameter θ_*l*_ across tiles, and $p_{i}=\sigma \left(x^{i}\cdot \bar{\theta }\right)$ is the predicted probability that *y*^*i*^ = 1 when considering the parameter mean $\bar{\theta }$. This penalty *R*({**θ**^**s**^}) can be interpreted as trying to compensate for high-confidence predictions (for which the term *p*_*i*_(1−*p*_*i*_) is small) by diminishing the pressure on ${\text{Var}}_{s}\left(\theta_{l}^{s_{l}}\right)$ to be small. As a result, samples **x**^*i*^'s for which the prediction will tend to be confident will be multiplied by weights θ_*l*_ that will display a relatively large variability across replica, which in turn will tend to reduce the degree of confidence.

This “confidence stabilization” effect raises the intriguing possibility that random tiling mitigates the weaknesses due to a model excessively high prediction confidence. The efficacy of *adversarial examples*, i.e., samples obtained with small perturbations resulting in intentional high-confidence misclassifications, is such a type of weakness that plagues several machine learning models (Goodfellow et al., 2014). Our analysis, suggests that random tiling should help immunize a model against this type of attacks, by preventing the model from being fooled with high confidence.

**Figure 7:**
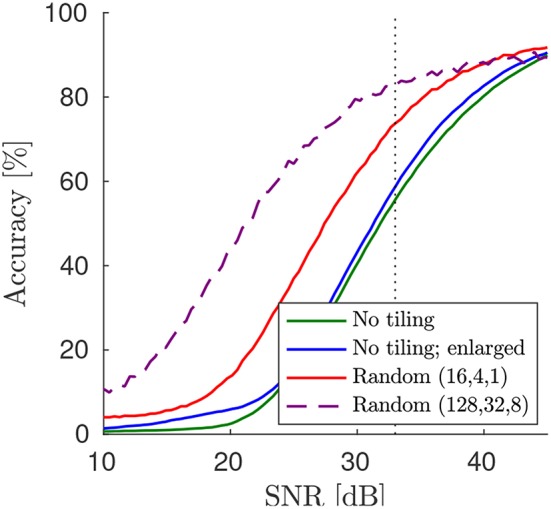
Tiling improves robustness to adversarial examples. Here, the random tiling network and the original (enlarged) ConvNet, all trained on the CIFAR-10 dataset (see Table 1), were tested on adversarial robustness. Note that using more kernel matrix replicas (here up to 128 for the first convolution) increases the robustness further.

We verify the theoretical prediction that random tiling increases the robustness to adversarial samples by using the Fast Gradient Sign Method (FSGM; Goodfellow et al., 2014) to attack a network trained on CIFAR-10 with max-pooling (see performance results in Table 1). In particular, we computed the accuracy drop from all correctly classified images in the test set, due to a perturbation by noise in the direction of the signed error gradient with strength ϵ (Goodfellow et al., 2014). Following Cisse et al. (2017), we computed the drop in accuracy as a function of the signal-to-noise ratio resulting from adversarial noise (see Figure 7). At a noise level corresponding to the threshold of human perception, ϵ ≈ 33 (according to Cisse et al., 2017), we find that random tiling reduces the gap to perfect adversarial robustness by around 41%. In comparison, other learning methods, such as Cisse et al. (2017) or enhancing training examples with adversarial gradients (Goodfellow et al., 2014) reduces the gap on CIFAR-10 by around 6% and 54%, respectively (using their baseline, compare to Cisse et al., 2017, Table 1). For other datasets, results are qualitatively similar, for instance, in case of CIFAR-100^+^ (strongly augmented) our original network reaches 25% at ϵ≈33, which improves to 62% with random tiling, and thus improves the gap by 49%. In comparison, the method in Cisse et al. (2017) improves the gap on CIFAR-100 only by 10 or 28%, respectively (depending on whether using enhanced training examples). While the networks used here are not the same as those used in Cisse et al. (2017), our results still suggest that random tiling significantly improves robustness, with no loss in performance or extra training examples.

A strategy to further improve robustness is to increase the number of tiles in the random tiling network. If we set *n*_*t*_ = (128, 32, 8) the network still trains fine with similar test error (see Figure 5). However, now robustness to adversarial attacks is significantly improved, reaching an accuracy of 83.97% for ϵ≈33 (see Figure 7; dashed line), which translates to a reduction of the gap to perfect robustness by 64%. Note that, although the *n*_*t*_ = (128, 32, 8) network has now about 20 times more convolutional weights than the original non-tiled network, it trains well and does not overfit (training error 15%) and, neglecting peripheral costs and assuming parallel execution of all analog array tiles in a layer, would execute a training epoch 128 times faster than the original network.

## 4. Discussion

### 4.1. Considerations About Algorithmic Generality and Specific Hardware Architectures

Here we proposed a modification of ConvNets that is amenable to a highly parallelizable implementation onto upcoming mixed analog-digital hardware.

Our considerations were mainly developed in the specific context of mixed analog-digital hardware, i.e., systems composed of digital computing unit and analog arrays (e.g., cross-point device arrays such as the RPUs described in Gokmen and Vlasov, 2016), that compute vector-matrix products in the analog domain with non-volatile memory elements, and convert results back into digital space where memory and compute in floating point precision is available. However, our proposal is valid for general systems where matrix-vector multiplications and rank-1 matrix updates can be performed in constant time (i.e., irrespective of the matrix size), and it is in fact agnostic to the implementational details of these operations.

Computing a matrix-*matrix* product in such systems, on the other hand, is typically relatively slow, since it is not computed in constant time but sequentially as a number of matrix-vector products. Clearly, this type of hardware has a speed advantage in regimes where weight matrices are large and the number of matrix-vector products is small, which is not the case for the first few convolutional layers (when mapped to matrix-matrix products with the *im*2*col*, or lowering operation).

Our algorithm can be interpreted as a method to represent convolutions in a way so that it can computed on multiple analog arrays in parallel without requiring memory movement of the weight matrices. This new representation harnesses the computational advantage and saturates the utilization of hardware architectures that implement constant-time matrix-vector products in non-volatile memory.

Our solution, RAPA-ConvNet, relies on the main idea of randomly dividing the computation load corresponding to one convolution operation among multiple independently and simultaneously trained kernel matrices. Remarkably, we find that this stochastic strategy yields no loss in accuracy, in particular, when utilizing a majority voting strategy for border line predictions. If executed on parallel analog arrays in a mixed analog-digital system, our architecture achieves a theoretical speedup that is linear in the number kernel replica used for the first convolution layer, and amounts to at least 16 times acceleration in our numerical experiments.

Note that this dramatic theoretical acceleration factor assumes that the run time of the system is not limited by the memory operations and computations on the digital part. Whether this is a reasonable assumption depends on additional factors, such as the concrete hardware implementation and the (noise) specifications of the analog part, all of which are beyond the scope of the current paper, which focuses on the algorithmic and functional aspect. However, since the central step of our algorithm consists in randomly assigning rows of the input data to different RPU arrays, the main computational overhead is due to the need of re-shuffling the *n*_*p*_ row-indices for each image, which can be done in linear time in the digital domain. Thus, the digital overhead is quantifiably small, in particular considering that the indices re-shuffling operation could be relaxed to re-shuffling only every few images with likely no impact on accuracy.

In this paper our goal was to propose RAPA-ConvNets, and provide algorithmic analysis and verification of the method. Therefore we focused on simulated analog RPU arrays in the ideal situation, where matrix-vector products could be computed noiselessly at floating point precision. This is clearly not realistic in an analog setting, due to the inaccuracies plaguing individual device elements, cycle-to-cycle update noise, update asymmetries, as well as limited resolution and bounded range of the analog-digital converters. These inaccuracies are in fact known to impact the training performance, as extensively analyzed in previous work (Gokmen et al., 2017). RAPA-ConvNets do not solve the learning difficulties due to such inaccuracies introduced by an analog computing implementation of ConvNets. Our work here instead focuses on the solution of a fundamental algorithmic limitation faced by the use of analog arrays for ConvNets. Our modified ConvNet architecture in particular solves a bottleneck in the run time scaling law of analog arrays, allowing for accelerated training through parallelization. To however test whether non-idealities of the analog compute would impact our conclusions, we performed additional simulations using an RPU base line model as described in Rasch et al. (2019) and found that results are very similar in the case of more realistic RPU simulations. We thus conclude that our RAPA-ConvNets does not add any qualitative different requirements on the specification of the analog array device elements beyond those discussed in previous work, in e.g.,Gokmen et al. (2017).

### 4.2. Empirical Verification of RAPA-ConvNets

We studied and validated the principles of our architecture in a small standard ConvNet. However, we expect the tiling architecture to be applicable also to larger ConvNets (e.g., Krizhevsky et al., 2012), because they generally successively reduce the spatial size with depth through pooling (Gu et al., 2018) and thus have a similar pattern of the amount of compute per layer as our example network (Figure 1). For instance, an efficient tiling of the architecture in Krizhevsky et al. (2012) would be *n*_*t*_ = (17, 4, 1, 1, 1). This would achieve perfect load-balancing across the 5 conv layers on analog arrays. Note that, if set up in this way, the whole network (including the fully connected layers) can additionally be pipelined across image batches (Ben-Nun and Hoefler, 2018), because the duration of computation would be identical for each of the conv layers (irrespective of the different filter sizes and number of channels).

There are many different approaches to accelerating deep learning using current hardware (Ben-Nun and Hoefler, 2018). Our approach is motivated by the constraints of mixed-analog digital hardware and the desire to emphasize its advantages. In our tiling approach, although the total amount of compute in the network is kept constant (contrary to e.g., methods that perforate the loop Figurnov et al., 2016, or use low-rank approximations or low precision weights, reviewed in Gu et al., 2018), the number of updates per weight is nevertheless reduced, which might generally affect learning curves. In our experiments, when increasing the number of tiles per layer to more than 128, weight update becomes too scarce, and the final performance indeed drops to a level that majority voting does not seem to rescue. To what degree additional techniques, such as learning rate adjustment or training time increase, could recover the performance drop for a large number of tiles, is subject of future research.

### 4.3. Comparison of Random Tiling to Other Tiling Methods

We found that, compared to other tested tiling methods, random tiling generally yields superior performance, when employing majority voting. The run time increase during inference due to majority voting could be minimized by implementing this mechanism only in the case of “uncertain” output predictions (as judged from the magnitude of the softmax layer output). Moreover, majority voting could be implemented in an iterative fashion, such that the fast initial prediction of the first network evaluation could be progressively combined with increasing evaluations gradually contributing to the accuracy of the majority vote. This mechanism can be fine tuned to trade-off prediction time and energy consumption with accuracy, similarly to what was for instance proposed in Mart́ı et al. (2016).

We found that other tiling methods improved when using strong augmentation techniques. This is to be expected, because the data augmentation shuffles the image in space and thus mirrors the random-sampling of the RAPA-ConvNet. As a result, the other tiling methods with fixed spatial mapping of image patches to tiles are better regularized and behave more similar to the random tiling network. Thus, strong data augmentation can to some degree reduce the need for randomly distributing image patches.

Among the alternative tiling methods examined, the alternating tiling method yielded lowest test error. Thus, it seems that a fixed local spatial relation of patches per tile distribution (that is approximately translation invariant across the image plane) can be advantageous. However, we found that in many simulations alternating tiling did not converge, and only came close or matched random tiling (with majority vote) in case of strong data augmentation and mixed pooling. Clearly, the self-regularization property of the random tiling method has advantages, in particular, when data is limited or data augmentation is not available. Moreover, it is not clear how to use the alternating method when tile numbers increase in an arbitrary (non-quadratic) fashion.

Finally, only for RAPA-ConvNets it is possible to reduce the tiled network to the non-tiled architecture, although performance is still somewhat impaired compared to training the original ConvNet directly. However, the alternative tiling methods that we tested caused performance of the reduced model to deteriorate to unacceptable levels.

### 4.4. Self-Regularization by Random Assignments

Besides our empirical verification of the RAPA-ConvNets, we also provide a theoretical analysis of our algorithm that explains its properties by connecting the random assignment across tiles with an implicit form of regularization, and, additionally, reveals a “confidence stabilization” effect resulting in increased robustness toward adversarial attacks.

Several regularization procedures based on randomization have been proposed in the literature: dropout and dropconnect are popular recent ones, and see Gu et al. (2018) for a recent review. Our finding that randomly splitting convolutions among several parallel tiles has a regularization effect is thus in line with this body of work. However, randomness in these regularization methods is typically restricted to the training phase, whereas the network architecture is fixed during testing. In contrast, because in our case the main goal of the randomization procedure is to speed up the computation through parallelization, random tiling is carried out both a training and at test time.

It has been found recently, although in a different context, that some forms of randomness during testing are indeed well suited for mitigating adversarial effects (Xie et al., 2017), which is similar to our finding. However, while the authors randomize only on the input level (image resizing or random padding), our architecture has builtin randomness in the convolutional layer, so that no change in the input images needs to be made to achieve the adversarial robustness.

### 4.5. Conclusion

Here, we evaluated a modified ConvNet architecture, that shows accelerated run time benefits when employed on upcoming hardware systems that can perform vector-matrix products in constant time with analog arrays. We found that the algorithmic modifications necessary to parallelize the convolution operation result in no appreciable loss in training performances compared to the original network. Furthermore, we found that random assignment of the compute to replicated kernel matrices have added advantages, such as improved accuracy by majority voting, adversarial robustness, and self-regularization. Our investigation thus suggests to revise the pessimistic notion that mixed analog-digital hardware cannot be used to accelerate ConvNets.

Finally, an interesting future research direction is how the performance of RAPA ConvNets could be further improved by increasing the convolution filter size or the number of filters per layer. Remarkably, this type of modifications, which are generally avoided on GPUs for reasons of efficiency, would not alter the overall run time on upcoming mixed analog-digital hardware technology.

## Data Availability

The datasets analyzed for this study can be found in the respective references as given in the main text.

## Author Contributions

MRa, TG, and MRi conceived the original ideas. MRa implemented and ran the simulations. MRi developed the theoretical analysis. MRa, TG, MRi, and WH analyzed and interpreted results and revised the manuscript. MRa and MRi drafted the manuscript.

### Conflict of Interest Statement

All authors were employed by company IBM.

## Footnotes

^1^We used the “Full” network from the Caffe cifar10 examples, except changing the sigmoid activations to ReLU and ensuring that pooling is done after the activation function.
